# State Public Assistance Spending and Survival Among Adults With Cancer

**DOI:** 10.1001/jamanetworkopen.2023.32353

**Published:** 2023-09-05

**Authors:** Justin M. Barnes, Kenton J. Johnston, Kimberly J. Johnson, Fumiko Chino, Nosayaba Osazuwa-Peters

**Affiliations:** 1 Department of Radiation Oncology, Washington University School of Medicine, St Louis, Missouri; 2 General Medical Sciences Division, Department of Medicine, Washington University School of Medicine, St Louis, Missouri; 3 Brown School, Washington University in St Louis, St Louis, Missouri; 4 Department of Radiation Oncology, Affordability Working Group, Memorial Sloan Kettering Cancer Center, New York, New York; 5 Department of Head and Neck Surgery & Communication Sciences, Duke University School of Medicine, Durham, North Carolina; 6 Department of Population Health Sciences, School of Medicine, Duke University, Durham, North Carolina; 7 Duke Cancer Institute, Duke University, Durham, North Carolina

## Abstract

**Importance:**

Social determinants of health contribute to disparities in cancer outcomes. State public assistance spending, including Medicaid and cash assistance programs for socioeconomically disadvantaged individuals, may improve access to care; address barriers, such as food and housing insecurity; and lead to improved cancer outcomes for marginalized populations.

**Objective:**

To determine whether state-level public assistance spending is associated with overall survival (OS) among individuals with cancer, overall and by race and ethnicity.

**Design, Setting, and Participants:**

This cohort study included US adults aged at least 18 years with a new cancer diagnosis from 2007 to 2013, with follow-up through 2019. Data were obtained from the Surveillance, Epidemiology, and End Results program. Data were analyzed from November 18, 2021, to July 6, 2023.

**Exposure:**

Differential state-level public assistance spending.

**Main Outcome and Measure:**

The main outcome was 6-year OS. Analyses were adjusted for age, race, ethnicity, sex, metropolitan residence, county-level income, state fixed effects, state-level percentages of residents living in poverty and aged 65 years or older, cancer type, and cancer stage.

**Results:**

A total 2 035 977 individuals with cancer were identified and included in analysis, with 1 005 702 individuals (49.4%) aged 65 years or older and 1 026 309 (50.4%) male. By tertile of public assistance spending, 6-year OS was 55.9% for the lowest tertile, 55.9% for the middle tertile, and 56.6% for the highest tertile. In adjusted analyses, public assistance spending at the state-level was significantly associated with higher 6-year OS (0.09% [95% CI, 0.04%-0.13%] per $100 per capita; *P* < .001), particularly for non-Hispanic Black individuals (0.29% [95% CI, 0.07%-0.52%] per $100 per capita; *P* = .01) and non-Hispanic White individuals (0.12% [95% CI, 0.08%-0.16%] per $100 per capita; *P* < .001). In sensitivity analyses examining the roles of Medicaid spending and Medicaid expansion including additional years of data, non-Medicaid spending was associated with higher 3-year OS among non-Hispanic Black individuals (0.49% [95% CI, 0.26%-0.72%] per $100 per capita when accounting for Medicaid spending; 0.17% [95% CI, 0.02%-0.31%] per $100 per capita Medicaid expansion effects).

**Conclusions and Relevance:**

This cohort study found that state public assistance expenditures, including cash assistance programs and Medicaid, were associated with improved survival for individuals with cancer. State investment in public assistance programs may represent an important avenue to improve cancer outcomes through addressing social determinants of health and should be a topic of further investigation.

## Key Points

**Question:**

Is state public assistance spending associated with overall survival (OS) in individuals with cancer?

**Findings:**

In this cohort study using data from 2 035 977 individuals with cancer in the Surveillance, Epidemiology and End Results database, public welfare spending was associated with higher 6-year OS overall and for non-Hispanic Black and non-Hispanic White subgroups.

**Meaning:**

These findings suggest that investment in state public welfare programs, including Medicaid and cash assistance programs for socioeconomically disadvantaged individuals, may improve cancer outcomes.

## Teaser

This cohort study assesses associations of state-level public assistance spending with overall survival in individuals with cancer overall and by race and ethnicity.

## Introduction

Social determinants of health, including health care access, socioeconomic status, and other socioenvironmental conditions and stressors, are significant contributors to adverse cancer outcomes in some populations, particularly for racial and ethnic minorities, including Hispanic and non-Hispanic Black populations.^1,2,3,4^ As evidenced by decreased cancer mortality due Medicaid expansion,^5,6,7,8,9^ federal- and state-funded programs for socioeconomically disadvantaged individuals may be poised to directly address some of the underlying social determinants of health affecting patients with cancer. This may increase access to treatment or a patient’s ability to complete treatment in a timely manner, ultimately improving outcomes.^10,11,12^ One potential but largely unexplored avenue to achieving equity in cancer outcomes is investment in public assistance programs.

Public assistance spending is a broad spending category accounting for approximately 20% of state-level expenditures in recent years.^13^ It encompasses programs providing assistance and services to individuals with lower socioeconomic status and includes a share of Medicaid spending, which comprises most of the expenditures, and programs that provide cash assistance for individuals (eg, Temporary Assistance for Needy Families [TANF] and Supplemental Security Income [SSI]).^13^ Eligibility criteria, often based on income and financial resources, and amounts of assistance provided can vary by state. Prior research has reported that public assistance spending was not significantly associated with overall cancer mortality rates but was significantly associated with lower lung cancer mortality rates.^14,15^ However, it is unclear whether public assistance spending is associated with survival times for individuals with cancer or improvements in outcomes among individuals from racial and ethnic minority groups. We hypothesized that higher public assistance expenditures would be associated with improved overall survival (OS) for individuals with cancer, particularly for individuals from racial and ethnic minority groups.

## Methods

This cohort study was not human participants research, so institutional review board oversight and informed consent were not required. Adults aged 18 years and older with a new cancer diagnosis from 11 states from 2007 to 2019 were identified in the Surveillance, Epidemiology, and End Results (SEER) program database and included in our study.^16^ All cancer sites were included. The outcome of interest was OS, which has been used commonly in analyses of survival among patients with cancer in relation to state-level policies and which we felt was more clinically relevant than cancer-specific survival (CSS); however, CSS was assessed in sensitivity analyses.^6,8,17,18,19^ Annual (time-variant, linked by year of cancer diagnosis) state expenditure data for each of the 50 US states and the District of Columbia, as reported in dollars per capita, were obtained from the US Census Bureau, which conducts comprehensive, nationwide government financial surveys of all 50 states and the District of Columbia.^13^ Specific components of state public assistance expenditures, which included both state and federal contributions, are based on codes included in the US Census Bureau public welfare expenditures category (eTable 1 in Supplement 1).^20^ Covariates were selected a priori and included age, race, ethnicity, sex, metropolitan residence, county-level income, state fixed effects, state-level poverty (based on American Community Survey data from the US Census Bureau),^21^ state-level age (percentage of populated aged >65 years, based on estimates from the American Community Survey),^22^ cancer type, and cancer stage. Race and ethnicity information was derived from a variety of sources^23,24^ and classified as Hispanic, non-Hispanic Black, non-Hispanic White, and non-Hispanic other (eg, Asian, Alaska Native, American Indian, and Pacific Islander). Note that public insurance is a component of public assistance expenditures. Additionally note that county-level information has recently been suppressed by SEER, precluding utilization of other county-level covariates. For exploratory analyses, we collected state spending data on total Medicaid expenditures, TANF expenditures, and SSI beneficiary expenditures from the National Association of State Budget Officers, US Department of Health and Human Services, and Social Security Administration, respectively.^25,26,27^ All spending data are presented in dollars per capita and were inflation-adjusted to 2019 dollars.^28^

**Figure 1.**
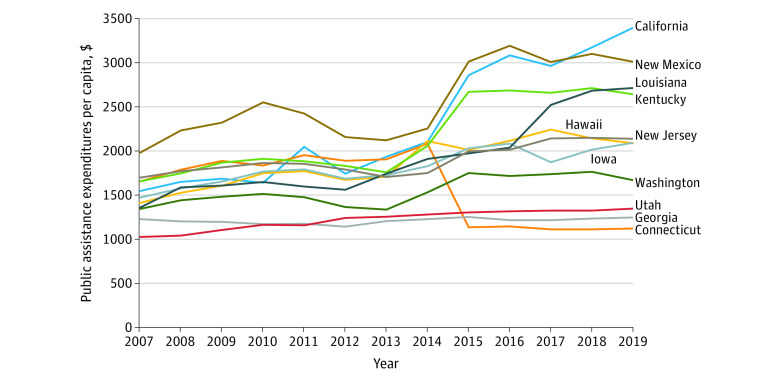
Temporal Trends in State Public Assistance Expenditures, 2007-2019 Note that public assistance expenditures were inflation-adjusted to 2019 dollars.

For our primary analyses, we limited the data to patients diagnosed in 2007 to 2013, since public assistance expenditures increased after 2014 following the implementation of Medicaid expansion in many states (Figure 1).^29^ Follow-up through 2019 enabled analysis of 6-year OS. However, since Medicaid is an important component of public assistance spending, we conducted several sensitivity analyses. First, to also minimize impacts from early Medicaid expansion (2010-2011) in some states as well as the recession of 2008 and 2009, we conducted analyses only including patients diagnosed from 2004 to 2007; follow-up enabled assessment of 12-year OS. Second, we limited the analyses to patients diagnosed from 2015 (after the Patient Protection and Affordable Care Act [ACA] was enacted in 2014, with wash-in period of 1 year) to 2019; patients from Louisiana were excluded from these analyses due to Medicaid expansion in mid-2016. Finally, we analyzed the full sample of patients from 2007 to 2019 with 2 approaches: to account for state Medicaid expansion effects, we included state by post-2013 interaction terms; we also examined changes in survival from increased public assistance spending induced by Medicaid expansion (via an interaction term between state Medicaid expansion status, post-2013 status, and public assistance spending). For analyses including post-2013 data, we analyzed 3-year OS (note that the median follow-up for censored cases diagnosed in 2014-2019 was 2.5 years) (eMethods in Supplement 1).

### Statistical Analysis

We evaluated the association of OS and state-level assistance spending in univariate analyses using the Kaplan-Meier method, with spending data divided into tertiles for visual simplicity. Adjusted analyses comparing survival and continuous public assistance spending were conducted using regression with selected covariates, with the pseudo-observation approach facilitating analyses of associations with OS in a linear regression framework. Briefly, the pseudo-observation method enables estimation of a given observation’s contribution to a survival rate based on weighted differences in survival from Kaplan-Meier estimates with and without the given observation and assumes that censoring is independent of covariates (eMethods in Supplement 1).^30,31^ We used robust SEs clustered by state, given that the exposure, public assistance spending, was determined at the state level.^32^ Note that the use of this modeling approach was largely driven by proportional hazards models becoming not computationally feasible when implementing clustering with the large set of data (eMethods in Supplement 1). We conducted analyses overall and by race and ethnicity. We also examined county income and cancer site subgroups.

To identify potential components of public assistance expenditures that were particularly influential, we conducted exploratory analyses by age subgroups (<65 and ≥65 years), since the types of expenditures differ for these groups (eg, TANF expenditures more likely for younger families, SSI benefits more likely for retired persons). We also conducted analyses with models using various components of public assistance spending, namely TANF spending, SSI spending, and total Medicaid expenditures. Old Age, Survivors, and Disability Insurance spending (commonly known as *Social Security*) was not included, given that it is a federal program and benefits do not vary at the state level. While the SSI program is also a federal program, many states provide state-determined additional benefits.^33^ To account for cost of living differences across states, which could alter the impact of assistance programs, we conducted sensitivity analyses in which expenditures were adjusted by the cost of living index.^34^ Expanded regression models with interaction effects were used to determine whether public assistance spending was associated with changes in racial and ethnic disparities in survival. We considered 2-sided *P* < .05 statistically significant. We used R software version 4.1.0 (R Project for Statistical Computing) for data analysis. Data were analyzed from November 18, 2021, to July 6, 2023.

## Results

The total cohort included 3 881 067 individuals from 2007 to 2019, with 2 035 977 individuals diagnosed from 2007 to 2013 (eFigure 1 in Supplement 1). Approximately half the cohort was aged 65 years or older (1 005 702 individuals [49.4%]), male (1 026 309 individuals [50.4%]), and diagnosed with localized disease (954 828 individuals [46.9%]) (Table 1). There were 222 326 Hispanic individuals (10.9%), 206 043 non-Hispanic Black individuals (10.1%), 1 444 128 non-Hispanic White individuals (70.9%), and 163 480 individuals who identified as non-Hispanic other race (8.0%); 1 782 233 individuals (87.5%) were metropolitan residents. The most common cancer sites were breast (317 884 individuals [15.6%]), prostate (294 898 individuals [14.5%]), and lung and bronchus (242 414 individuals [11.9%]). A higher percentage of individuals who were excluded were aged 65 years or older, male, and had unknown stage at diagnosis or unstaged cancers (eTable 2 in Supplement 1). There was no statistically significant difference in public assistance spending per capita between the excluded individuals and the analytic sample (eTable 2 in Supplement 1). The mean (range) state public assistance spending over 2007 to 2013 was $1660 ($1027-$2552) per capita, with 33rd and 66th percentiles $1640 to $1760 (Table 1; eFigure 2 and eFigure 3 in Supplement 1). Data characteristics for individuals diagnosed from 2007 to 2019 were similar (eTable 3 and eTable 4 in Supplement 1).

**Table 1.** Characteristics of the Study Population, 2007-2013

Characteristic	State public assistance expenditures, No. (%)			
	Total (N = 2 035 977)	≤$1640 Per Capita (n = 652 176)	$1641-$1760 Per Capita (n = 720 506)	>$1760 Per Capita (n = 663 295)
Age, y				
18-39	114 970 (5.6)	38 372 (5.9)	40 643 (5.6)	35 955 (5.4)
40-54	390 836 (19.2)	129 181 (19.8)	136 336 (18.9)	125 319 (18.9)
55-64	524 469 (25.8)	172 208 (26.4)	182 453 (25.3)	169 808 (25.6)
≥65	1 005 702 (49.4)	312 415 (47.9)	361 074 (50.1)	332 213 (50.1)
Race and ethnicity				
Hispanic	222 326 (10.9)	36 116 (5.5)	100 134 (13.9)	86 076 (13)
Non-Hispanic Black	206 043 (10.1)	100 220 (15.4)	56 903 (7.9)	48 920 (7.4)
Non-Hispanic White	1 444 128 (70.9)	475 935 (73)	488 505 (67.8)	479 688 (72.3)
Non-Hispanic other^a^	163 480 (8)	39 905 (6.1)	74 964 (10.4)	48 611 (7.3)
Sex				
Male	1 026 309 (50.4)	335 667 (51.5)	361 107 (50.1)	329 535 (49.7)
Female	1 009 668 (49.6)	316 509 (48.5)	359 399 (49.9)	333 760 (50.3)
Residence				
Nonmetropolitan	253 744 (12.5)	105 730 (16.2)	70 659 (9.8)	77 355 (11.7)
Metropolitan	1 782 233 (87.5)	546 446 (83.8)	649 847 (90.2)	585 940 (88.3)
Median household county income (inflation adjusted)				
Quartile 1 (lowest)	492 382 (24.2)	214 329 (32.9)	138 791 (19.3)	139 262 (21)
Quartile 2	503 022 (24.7)	116 378 (17.8)	211 156 (29.3)	175 488 (26.5)
Quartile 3	460 920 (22.6)	186 148 (28.5)	151 566 (21)	123 206 (18.6)
Quartile 4 (highest)	579 653 (28.5)	135 321 (20.7)	218 993 (30.4)	225 339 (34)
Marital status				
Not married	832 172 (40.9)	260 640 (40)	298 603 (41.4)	272 929 (41.1)
Married	1 203 805 (59.1)	391 536 (60)	421 903 (58.6)	390 366 (58.9)
Stage at diagnosis				
Localized	954 828 (46.9)	308 563 (47.3)	334 495 (46.4)	311 770 (47)
Regional	445 085 (21.9)	144 247 (22.1)	158 005 (21.9)	142 833 (21.5)
Distant	510 463 (25.1)	164 493 (25.2)	180 950 (25.1)	165 020 (24.9)
Unknown or unstaged	125 601 (6.2)	34 873 (5.3)	47 056 (6.5)	43 672 (6.6)
Cancer site				
Breast	317 884 (15.6)	101 831 (15.6)	112 600 (15.6)	103 453 (15.6)
Cervical	19 819 (1)	6390 (1)	7315 (1)	6114 (0.9)
Colorectal	178 295 (8.8)	57 048 (8.7)	64 477 (8.9)	56 770 (8.6)
Head and neck	66 381 (3.3)	22 218 (3.4)	23 094 (3.2)	21 069 (3.2)
Hodgkin lymphoma	12 110 (0.6)	4021 (0.6)	4157 (0.6)	3932 (0.6)
Kidney and renal pelvis	67 856 (3.3)	21 636 (3.3)	24 312 (3.4)	21 908 (3.3)
Leukemia	51 002 (2.5)	15 882 (2.4)	18 037 (2.5)	17 083 (2.6)
Liver	37 011 (1.8)	10 675 (1.6)	14 276 (2)	12 060 (1.8)
Lung and bronchus	242 414 (11.9)	81 044 (12.4)	83 511 (11.6)	77 859 (11.7)
Myeloma	28 707 (1.4)	9354 (1.4)	10 042 (1.4)	9311 (1.4)
Non-Hodgkin lymphoma	83 512 (4.1)	26 374 (4)	29 848 (4.1)	27 290 (4.1)
Other	212 300 (10.4)	66 449 (10.2)	76 231 (10.6)	69 620 (10.5)
Ovary	31 229 (1.5)	9832 (1.5)	11 237 (1.6)	10 160 (1.5)
Pancreas	55 276 (2.7)	17 134 (2.6)	19 795 (2.7)	18 347 (2.8)
Prostate	294 898 (14.5)	102 304 (15.7)	100 623 (14)	91 971 (13.9)
Skin	80 048 (3.9)	24 279 (3.7)	28 991 (4)	26 778 (4)
Stomach	32 903 (1.6)	9515 (1.5)	12 383 (1.7)	11 005 (1.7)
Testis	13 937 (0.7)	4362 (0.7)	5096 (0.7)	4479 (0.7)
Thyroid	63 897 (3.1)	18 778 (2.9)	22 101 (3.1)	23 018 (3.5)
Urinary bladder	79 062 (3.9)	23 661 (3.6)	28 120 (3.9)	27 281 (4.1)
Uterus	67 436 (3.3)	19 389 (3)	24 260 (3.4)	23 787 (3.6)
State-level characteristics, mean (SD)				
Poverty, %	14.64 (2.79)	15.55 (2.16)	14.84 (2.26)	13.54 (3.43)
Aged ≥65 y, %	12.27 (1.34)	11.50 (1.31)	12.20 (1.24)	13.11 (0.93)
Spending per capita, $	1660.1 (258.8)	1363.2 (174.5)	1686.9 (40.1)	1922.9 (132.0)

^a^ Includes Asian, Alaska Native, American Indian, and Pacific Islander.

**Figure 2.**
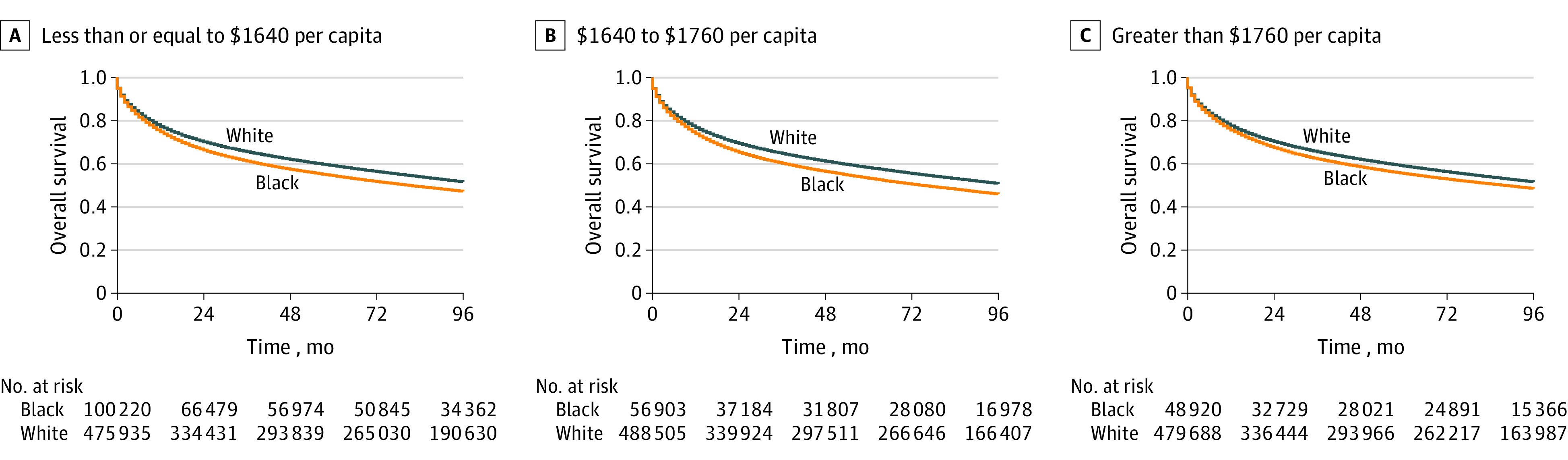
Overall Survival by State Public Assistance Expenditure Tertiles for Non-Hispanic White and Non-Hispanic Black Individuals Public assistance expenditures given were inflation-adjusted to 2019 dollars.

In Kaplan-Meier analyses, 6-year OS was 55.9% (95% CI, 55.8% to 56.1%) for the lowest spending tertile (≤$1640 per capita), 55.9% (95% CI, 55.8% to 56.0%) for the middle spending tertile ($1641 to $1760), and 56.6% (95% CI, 56.4% to 56.7%) for the highest spending tertile (>$1760/capita). By race and ethnicity, 6-year OS was 60.0% (95% CI, 59.5% to 60.5%) in the lowest spending tertile and 58.2% (95% CI, 57.9% to 58.5%) in the highest spending tertile for Hispanic individuals, 51.7% (95% CI, 51.4% to 52.0%) in the lowest spending tertile and 52.9% (95% CI, 52.5% to 53.4%) in the highest spending tertile for non-Hispanic Black individuals, 56.3% (95% CI, 56.2% to 56.5%) in the lowest spending tertile and 56.2% (95% CI, 56.1% to 56.4%) in the highest spending tertile for non-Hispanic White individuals, and 58.2% (95% CI, 57.7% to 58.7%) in the lowest spending tertile and 60.7% (95% CI, 60.3% to 61.2%) in the highest spending tertile for individuals who identified as non-Hispanic other race (Figure 2; eFigure 4 in Supplement 1).

In our primary analyses examining 6-year OS in individuals diagnosed from 2007 to 2013, higher public assistance spending was associated with higher OS overall (0.09% [95% CI, 0.04% to 0.13%] per $100 per capita; *P* < .001) (Table 2). State fixed effects from the model are given in eTable 5 in Supplement 1. The largest improvements in OS associated with public assistance spending were among non-Hispanic Black individuals (0.29% [95% CI, 0.07% to 0.52%] per $100 per capita; *P* = .01) and non-Hispanic White individuals (0.12% [95% CI, 0.08% to 0.16%] per $100 per capita; *P* < .001), in contrast to no significant associations among other racial and ethnic subgroups (Table 2). In expanded models with public assistance spending by race and ethnicity interaction terms, there was no significant attenuation of disparities in OS for racial and ethnic minority groups compared with non-Hispanic White individuals in any of our analyses.

**Table 2.** Associations of Public Assistance Spending and 6-Year Overall Survival, 2007-2013

Race and ethnicity	Estimate (95% CI), %^a^	*P* value
Overall	0.09 (0.04 to 0.13)	<.001
Hispanic	0.05 (−0.03 to 0.13)	.22
Non-Hispanic Black	0.29 (0.07 to 0.52)	.01
Non-Hispanic White	0.12 (0.08 to 0.16)	<.001
Non-Hispanic other^b^	−0.01 (−0.16 to 0.15)	.92

^a^ Estimates are scaled to change in survival per $100 per capita. Estimates greater than 0 suggest that higher spending is associated with improved survival. Models were adjusted for age, race and ethnicity (except race and ethnicity subgroup analyses), sex, metropolitan residence, marital status, county-level income, state fixed effects, state-level poverty and percentage of individuals aged 65 years or older, cancer type, and cancer stage.

^b^ Includes Asian, Pacific Islander, American Indian, and Alaska Native.

In our sensitivity analyses, results were similar when analyzing patients diagnosed from 2004 to 2007, including significantly higher 12-year OS associated with higher public assistance expenditures for the overall population (0.37% [95% CI, 0.15% to 0.59%] per $100 per capita), as well as in subgroup analyses of Hispanic (0.39% [95% CI, 0.09% to 0.68%] per $100 per capita), non-Hispanic Black (0.67% [95% CI, 0.25% to 1.08%] per $100 per capita), and non-Hispanic White (0.36% [95% CI, 0.13% to 0.58%] per $100 per capita) populations (Table 3). When limiting the analyses to 2015 to 2019, public assistance spending was associated with reduced OS for the Hispanic population (−0.09% [95% CI, −0.15% to −0.03%] per $100 per capita); however, after accounting for Medicaid spending, public assistance spending was associated with higher OS for non-Hispanic Black individuals (0.49% [95% CI, 0.26% to 0.72%] per $100 per capita) with no significant association for Hispanic individuals (0.17% [95% CI, −0.15% to 0.49%] per $100 per capita). When examining 3-year OS among patients diagnosed from 2007 to 2019 after accounting for state Medicaid expansion, public assistance spending was associated with higher OS for the Hispanic (0.06% [95% CI, 0.00% to 0.12%] per $100 per capita; *P* = .04) and non-Hispanic Black (0.17% [95% CI, 0.02% to 0.31%] per $100 per capita; *P* = .02) subgroups but not the overall sample (0.01% [95% CI, −0.06% to 0.09%] per $100 per capita) or the non-Hispanic White (0.01% [95% CI, −0.05% to 0.07%] per $100 per capita) or non-Hispanic other (−0.02% [95% CI, −0.52% to 0.27%] per $100 per capita) subgroups. Public assistance spending induced by Medicaid expansion was associated with higher 3-year OS for non-Hispanic White individuals (4.24% [95% CI, 0.99% to 7.49%] per $100 per capita). Evaluation of CSS yielded similar results (eTable 6 in Supplement 1).

**Table 3.** Associations of Public Assistance Spending and Survival in Sensitivity Analyses

Outcome, by public assistance spending and race and ethnicity	Estimate (95% CI), %^a^	*P* value
12-y OS by total spending (2004-2007)		
Overall	0.37 (0.15 to 0.59)	.001
Hispanic	0.39 (0.09 to 0.68)	.01
Non-Hispanic Black	0.67 (0.25 to 1.08)	.002
Non-Hispanic White	0.36 (0.13 to 0.58)	.002
Non-Hispanic other^b^	0.06 (−0.05 to 0.18)	.28
3-y OS by total spending (2015-2019)^c^		
Overall	−0.01 (−0.03 to 0)	.14
Hispanic	−0.09 (−0.15 to −0.03)	.005
Non-Hispanic Black	−0.06 (−0.16 to 0.03)	.20
Non-Hispanic White	0 (−0.02 to 0.02)	.87
Non-Hispanic other^b^	−0.07 (−0.15 to 0.01)	.08
3-y OS after accounting for Medicaid spending (2015-2019)^c^^,^^d^		
Overall	−0.21 (−0.43 to 0.01)	.06
Hispanic	0.17 (−0.15 to 0.49)	.29
Non-Hispanic Black	0.49 (0.26 to 0.72)	<.001
Non-Hispanic White	−0.32 (−0.6 to −0.04)	.03
Non-Hispanic other^b^	−0.13 (−0.52 to 0.27)	.54
3-y OS after accounting for state Medicaid expansion effects (2007-2019)^e^		
Overall	0.01 (−0.06 to 0.09)	.71
Hispanic	0.06 (0 to 0.12)	.04
Non-Hispanic Black	0.17 (0.02 to 0.31)	.02
Non-Hispanic White	0.01 (−0.05 to 0.07)	.76
Non-Hispanic other^b^	−0.02 (−0.08 to 0.05)	.64
3-y OS with spending induced by the ACA Medicaid expansion (2007-2019)^f^		
Overall	3.35 (−0.21 to 6.91)	.07
Hispanic	1.56 (−4.24 to 7.36)	.60
Non-Hispanic Black	1.37 (−1.27 to 4.01)	.31
Non-Hispanic White	4.24 (0.99 to 7.49)	.01
Non-Hispanic other^b^	−1.43 (−7.33 to 4.47)	.63

Abbreviations: ACA, Patient Protection and Affordable Care Act; OS, overall survival.

^a^ Estimates are scaled to change in survival per $100 per capita. Models were adjusted for age, race and ethnicity (except race and ethnicity subgroup analyses), sex, metropolitan residence, marital status, county-level income, state fixed effects, state-level poverty and percentage of individuals aged 65 years and older, cancer type, and cancer stage.

^b^ Includes Asian, Pacific Islander, American Indian, and Alaska Native.

^c^ Louisiana was excluded from study population due to Medicaid expansion in mid-2016.

^d^ Models additionally included state-level Medicaid spending as a covariate.

^e^ Models were expanded to include covariates accounting for Medicaid expansion effects, which were captured by the inclusion of state by post-2014 interaction effects, which allow for state-level heterogeneity in expansion policies.

^f^ Analyses were limited to individuals aged 18 to 64 years, the population potentially eligible for Medicaid coverage under the expansions. The coefficient of interest corresponds to a state Medicaid expansion status (expansion vs nonexpansion state), post-2014 status (2007-2013 vs 2014-2019), and public assistance spending.

In exploratory analyses by age, the associations of improved OS with higher public assistance expenditures were more pronounced for individuals aged 18 to 64 years among the overall cohort and Hispanic, non-Hispanic Black, and non-Hispanic White subgroups, and public assistance spending was associated with lower OS associated for non-Hispanic Black individuals age 65 years or older (eTable 7 in Supplement 1). There were no clear patterns by cancer site subgroups or county-level income subgroups (eTable 8 in Supplement 1). When examining individual components of public assistance spending, higher Medicaid expenditures and other expenditures were associated with improved survival in some subgroups (eTable 7 in Supplement 1). Higher TANF spending was associated with lower OS for individuals aged 18 to 64 years cohort overall as well as the non-Hispanic White and non-Hispanic other subgroups. Patterns were similar when adjusting expenditures for cost of living (eTable 9 in Supplement 1). After adjusting for Medicaid eligibility levels, Medicaid expenditures were not associated with increased survival (eTable 9 in Supplement 1).

## Discussion

The findings of this cohort study suggest that higher state public assistance spending was associated with higher OS for individuals with cancer. After accounting for Medicaid spending or Medicaid expansion, survival improvements associated with public assistance spending were largely limited to non-Hispanic Black individuals. However, there was no significant attenuation in the survival disparity between non-Hispanic Black and non-Hispanic White subgroups with public assistance spending.

To our knowledge, these data are the first to examine the association of public assistance with survival among individuals with cancer, particularly by race and ethnicity. Other studies have shown associations of the ratio of social services spending (cash assistance, public health, transportation, education, and housing expenditures, among others) to health services spending (Medicare and Medicaid expenditures) with significantly lower lung cancer mortality.^15^ Our data, which focus on public assistance expenditures, defined as cash assistance and health insurance programs targeted to low-income individuals, suggest a similar pattern for survival from all cancers combined. In another study, public assistance spending was not significantly associated with population-level overall cancer mortality.^14^

Improved outcomes associated with increased state investment in public assistance could be due to altering the social determinants of health, which include, among others, socioeconomic conditions and health care access.^2^ Public assistance spending, through cash assistance programs to low-income individuals as well as support of health care access via Medicaid programs, should improve a person’s socioeconomic condition, access to quality health care, and ability to pay for care.^1,2^ Investing in nonmedical interventions to address social determinants of health and improve health is not a new idea. Food insecurity and housing insecurity, for example, have been associated with lower rates of completing recommended treatments and worse outcomes among individuals with cancer.^3,35,36^ Financial toxic effects are also associated with worse cancer outcomes.^37,38^ Recent interventions have specifically targeted food insecurity and have included insurance policy–based and clinic-based food assistance programs providing access to food pantries, food vouchers, and meal and grocery delivery.^39,40,41^ Findings from a prospective randomized study of various clinic-based food insecurity interventions for individuals undergoing cancer treatment suggested that food vouchers can decrease food insecurity and increase treatment completion rates,^41^ which have been associated with cancer outcomes.^42,43^ Hence, state-administered assistance programs may improve cancer outcomes by addressing unmet social needs, perhaps enabling individuals to better access needed cancer treatment and reduce financial toxic effects. Our data showing an association between public assistance spending and survival for individuals with cancer suggest that state public assistance expenditures are an investment in the health of the population.^1,2^

In sensitivity analyses, non-Medicaid public assistance spending was associated with higher survival for non-Hispanic Black individuals (ie, after adjusting for Medicaid spending or adjusting for Medicaid expansion effects). However, the role of Medicaid spending within public assistance expenditures was less clear for other races and ethnicities. While Medicaid expansion–induced public assistance spending was associated with higher survival for non-Hispanic White individuals with cancer, non-Medicaid public assistance spending (ie, public assistance spending after adjusting for Medicaid spending, but not after adjusting for Medicaid expansion effects) was associated with worse survival. Additionally, public assistance spending from 2015 to 2019, which was substantially impacted by spending related to Medicaid expansion, was associated with worse survival among Hispanic individuals with cancer, although, like the non-Hispanic Black subgroup, there were improvements in survival associated with public assistance spending after accounting for state Medicaid expansion effects. Together, these results suggest that different public assistance programs may affect populations differently. Specifically, improved outcomes associated with Medicaid-related public assistance spending may be limited to non-Hispanic White individuals, which is further consistent with our analyses of spending components showing associations of Medicaid spending and higher survival for non-Hispanic White individuals aged 18 to 64 years and consistent with prior reports demonstrating improved Medicaid expansion-associated cancer outcomes for non-Hispanic White individuals but mixed results for other races and ethnicities.^6,7,8^ Other (non-Medicaid) public assistance programs may be important for improving cancer outcomes for racial and ethnic minority groups.

While Medicaid spending and other (ie, not TANF, Medicaid, or SSI) spending were associated with improved outcomes for some subgroups, the specific components of public assistance spending driving better cancer outcomes for the overall cohort and other races and ethnicities are unclear in the setting of inconsistencies across our overall and age subgroup analyses. One interpretation of the lack of consistency is that investment in any public assistance program may improve outcomes, where state-level priorities of those programs may either be less important or be in-line with the needs of its population. Alternatively, the lack of important studied components may indicate that other state-run programs, or perhaps subcomponents of SSI, TANF, or Medicaid expenditures, are at play.

Prior studies have found evidence for disproportionate allocation of TANF funds away from marginalized populations, risking exacerbation of existing disparities.^44,45^ Consistent with these reports, we found that higher TANF spending was associated with worse outcomes for individuals aged 18 to 64 years. Hence, our data likely indicate that there has been suboptimal allocation of TANF funds that has ultimately adversely impacted patient outcomes, which may require policy solutions to address, such as removing work requirements or diverting funds toward basic cash assistance.^46,47,48^

### Limitations

This study has limitations, particularly regarding its retrospective nature and the presence of potential factors outside of the studied covariates that could confound our findings. The SEER database only includes a small number of states, which may not be representative of all states from a policy and spending standpoint. Furthermore, while most public assistance is provided at the state level (>90%), there is considerable variation across states in local government administration of public assistance benefits and spending on public assistance (eg, spending ranges from <1% of public assistance direct expenditures at the local level in Kentucky up to 16% in California), which is not accounted for in our analyses.^49^ Additionally, while we account for state fixed effects, there are a variety of other state factors that could contribute to our findings. While we suspect that increased access to cancer treatment contributes to the present findings, this was not examined due to limited treatment information, and establishing the associations of intermediate outcomes with public assistance spending will be required to add additional credibility to our findings. Similarly, evolutions in practice patterns, particularly for patients with recurrent disease, were not accounted for. Additionally, public assistance spending is a broad category, and states prioritize certain public assistance programs differently; while we explored potential important components of this spending, the components underlying its association with cancer outcomes are unclear, and meaningful policy and spending changes cannot happen until these are clarified by future research.

## Conclusions

This cohort study found that public assistance spending was associated with improved OS for individuals with cancer. Additional state investment in public assistance programs and/or reallocating existing funds may represent important strategies to improve cancer outcomes and should be a topic of further study.

## Notes

Supplement 1.
eMethods.

eReferences.
**eTable 1.** Public Assistance Expenditure Components per US Government Public Welfare Definition **eTable 2.** Characteristics of Missing/Excluded Data, 2007-2013 **eTable 3.** Characteristics of the Study Population, 2007-2019 **eTable 4.** Characteristics of Missing/Excluded Data, 2007-2019 **eTable 5.** State Fixed Effects for Primary Analysis (Overall Cohort, 2007-2013) **eTable 6.** Sensitivity Analysis Evaluating Associations of Public Assistance Spending and 6-Year Cancer-Specific Survival (CSS), 2007-2013 **eTable 7.** Association of Components of Public Assistance Expenditures and 6-Year Overall Survival **eTable 8.** Subgroup Analyses by Cancer Site and County Income Level (Overall Cohort, 2007-2013) **eTable 9.** Sensitivity Analyses Adjusting for Cost of Living by State and for State Medicaid Eligibility Levels (2007-2013) **eFigure 1.** Flowchart for Derivation of Study Sample **eFigure 2.** Distribution of Public Welfare Spending Across Individuals From States Included in the Surveillance, Epidemiology, and End Results Program, 2007-2013 **eFigure 3.** Distribution of Public Welfare Spending Across Individuals From States Included in the Surveillance, Epidemiology, and End Results Program, 2007-2019 **eFigure 4.** Overall Survival by State Public Assistance Expenditures for Non-Hispanic Other and Hispanic Individuals

Supplement 2.
Data Sharing Statement
