# Supplementary material for: State Public Assistance Spending and Survival Among Adults With Cancer
Source: JAMA Netw Open. 2023 Sep 5;6(9):e2332353. doi: 10.1001/jamanetworkopen.2023.32353 (PMC10481229; doi:10.1001/jamanetworkopen.2023.32353)
Supplement: Supplement 1. — eMethods. eReferences. eTable 1. Public Assistance Expenditure Components per US Government Public Welfare Definition eTable 2. Characteristics of Missing/Excluded Data, 2007-2013 eTable 3. Characteristics of the Study Population, 2007-2019 eTable 4. Characteristics of Missing/Excluded Data, 2007-2019 eTable 5. State Fixed Effects for Primary Analysis (Overall Cohort, 2007-2013) eTable 6. Sensitivity Analysis Evaluating Associations of Public Assistance Spending and 6-Year Cancer-Specific Survival (CSS), 2007-2013 eTable 7. Association of Components of Public Assistance Expenditures and 6-Year Overall Survival eTable 8. Subgroup Analyses by Cancer Site and County Income Level (Overall Cohort, 2007-2013) eTable 9. Sensitivity Analyses Adjusting for Cost of Living by State and for State Medicaid Eligibility Levels (2007-2013) eFigure 1. Flowchart for Derivation of Study Sample eFigure 2. Distribution of Public Welfare Spending Across Individuals From States Included in the Surveillance, Epidemiology, and End Results Program, 2007-2013 eFigure 3. Distribution of Public Welfare Spending Across Individuals From States Included in the Surveillance, Epidemiology, and End Results Program, 2007-2019 eFigure 4. Overall Survival by State Public Assistance Expenditures for Non-Hispanic Other and Hispanic Individuals [file jamanetwopen-e2332353-s001.pdf]

Supplemental Online Content

Barnes JM, Johnston KJ, Johnson KJ, Chino F, Osazuwa-Peters N. State public assistance spending and survival among adults with cancer. *JAMA Netw Open*. 2023;6(9):e2332353. doi:10.1001/jamanetworkopen.2023.32353

eMethods.
eReferences.
eTable 1. Public Assistance Expenditure Components per US Government Public Welfare Definition
eTable 2. Characteristics of Missing/Excluded Data, 2007-2013
eTable 3. Characteristics of the Study Population, 2007-2019
eTable 4. Characteristics of Missing/Excluded Data, 2007-2019
eTable 5. State Fixed Effects for Primary Analysis (Overall Cohort, 2007-2013)
eTable 6. Sensitivity Analysis Evaluating Associations of Public Assistance Spending and 6-Year Cancer-Specific Survival (CSS), 2007-2013
eTable 7. Association of Components of Public Assistance Expenditures and 6-Year Overall Survival
eTable 8. Subgroup Analyses by Cancer Site and County Income Level (Overall Cohort, 2007-2013)
eTable 9. Sensitivity Analyses Adjusting for Cost of Living by State and for State Medicaid Eligibility Levels (2007-2013)
eFigure 1. Flowchart for Derivation of Study Sample
eFigure 2. Distribution of Public Welfare Spending Across Individuals From States Included in the Surveillance, Epidemiology, and End Results Program, 2007-2013
eFigure 3. Distribution of Public Welfare Spending Across Individuals From States Included in the Surveillance, Epidemiology, and End Results Program, 2007-2019
eFigure 4. Overall Survival by State Public Assistance Expenditures for Non-Hispanic Other and Hispanic Individuals

This supplemental material has been provided by the authors to give readers additional information about their work.

© 2023 Barnes JM et al. *JAMA Network Open.*

eMethods

Age as a covariate

We utilized state-level %age 65+ as a covariate in addition to the individual-level age since some spending policies included in the total public assistance spending amount are targeted primarily at those who are retired, and we wanted to account for such state differences that could potentially drive portions of public assistance spending.

Pseudo-observation approach for modeling overall survival

We estimated pseudo-observations, which can be integrated into a linear regression framework as the dependent variable. The selection of the modeling approach using linear regression with pseudo-observations was largely driven by our inability to utilize proportional hazards models, which were not computationally feasible with these very large sets of data, specifically when implementing clustering. Pseudo-observations are an estimate of each individual observation's contribution to the total X-year overall survival as measured in a Kaplan-Meier curve. The resultant individual-level information is then integrated into the linear regression model. The pseudo-observations are calculated by estimating:

(1) The X-year overall survival for the overall sample based on the Kaplan-Meier method
(2) For all $j \in (1, 2, \ldots, N)$, estimate the X-year overall survival for the sample excluding observation j
(3) the pseudo-observation for observation j is equal to the weighted difference between the two previously computed values: $(N)*\text{X-yr-OS}_{\text{full sample}} - (N-1)*\text{X-yr-OS}_{\text{sample excluding } j}$.[1]

Estimation was done using the jackknife function from the R prodlim package.

© 2023 Barnes JM et al. *JAMA Network Open.*

In the pseudo-observation approach, censoring is assumed to be independent of the risk and time of a survival event and independent of covariates.[1,2] This is in contrast to other time-to-event analytical methods, such as the proportional hazards model, which notably allows covariate-dependent censoring. For our study, the primary concern was that censoring was related to year of diagnosis and the length of follow-up time available; to circumvent this issue, our primary analyses utilized a 6-year overall survival endpoint and included cases diagnosed up to 2013 followed through 2019, enabling 6 full years of follow up. Note that public assistance spending was relatively stable from 2007 to 2013. However, since censoring is related to the year of diagnosis, which also defines (yearly) public assistance spending, which changes substantially after 2014, pseudo-observations were estimated separately for the cohort of observations from 2007-2013 and for the cohort of observations from 2014-2019.[1] However, limited follow-up in the 2014-2019 cohort required the use of 3-year OS rather than estimates at later time points. To assess the robustness of our analyses to potential bias introduced by these assumptions, we compared estimates from our primary approach to estimates from a Cox proportional hazards model on a restricted sample (see below), with concordant results.

Use of the pseudo-observation approach over use of proportional hazards models

Use of proportional hazards models was not computationally feasible when implementing clustering with the large set of data. However, we examined the robustness of our pseudo-observation approach by taking two random samples of 50,000 observations (2.5% of the total sample size) and applying our pseudo-observation approach and a proportional hazards model, both with state-level clustering and the same covariates. The directionality of the results and

© 2023 Barnes JM et al. *JAMA Network Open.*

precision of the estimates were extremely similar between the approaches (note that the precision of these estimates is, expectedly, less than the overall sample with resultant larger P-values given the much smaller sample size):

Random Sample 1:

Pseudo-observation: Estimate = 0.21, P=0.10

Proportional Hazards: Hazard Ratio = 0.990, P=0.16

Random Sample 2:

Pseudo-observation: Estimate = 0.08, P=0.38

Proportional Hazards: Hazard Ratio = 0.998, P=0.38

© 2023 Barnes JM et al. *JAMA Network Open.*

eReferences

1. Andersen PK, Pohar Perme M. Pseudo-observations in survival analysis. *Stat Methods Med Res*. 2010;19(1):71-99. doi:10.1177/0962280209105020
2. Mortensen LM, Hansen CP, Overvad K, Lundbye-Christensen S, Parner ET. The Pseudo-observation analysis of time-to-event data. example from the danish diet, cancer and health cohort illustrating assumptions, model validation and interpretation of results. *Epidemiol Methods*. 2018;7(1). doi:10.1515/em-2017-0015

© 2023 Barnes JM et al. *JAMA Network Open.*

eTable 1: Public assistance expenditure components per US government public welfare definition

Item Code	Description	Definition and examples
E74	Current Operations - Welfare, Vendor Payments for Medical Care	Public welfare payments made directly to private vendors for medical assistance and hospital or health care, including Medicaid (Title XIX), plus mandatory state payments to the Federal Government to offset costs of prescription drugs under Medicare Part D. Payments to vendors or the Federal Government must be made on behalf of low-income or means tested beneficiaries, or other medically qualified persons.
E75	Current Operations - Welfare, Vendor Payments for Other Purposes	Payments under public welfare programs made directly to private vendors (i.e., individuals or nongovernmental organizations furnishing goods and services) for services and commodities, other than medical, hospital, and health care, on behalf of low-income or other means-tested beneficiaries.
E77	Current Operations - Welfare Institutions	Provision, construction, and maintenance of nursing homes and welfare institutions owned and operated by a government for the benefit of needy persons (contingent upon their financial or medical need), and veterans.
E79	Current Operations - Welfare, Other	All expenditures for welfare activities not classified elsewhere (for example, expenditures supported by Federal Social Services Block Grant (Title XX) funds)
F77	Construction - Welfare Institutions	Provision, construction, and maintenance of nursing homes and welfare institutions owned and operated by a government for the benefit of needy persons (contingent upon their financial or medical need), and veterans.
F79	Construction - Welfare, Other	All expenditures for welfare activities not classified elsewhere (for example, expenditures supported by Federal Social Services Block Grant (Title XX) funds)
G77	Other Capital Outlay - Welfare Institutions	Provision, construction, and maintenance of nursing homes and welfare institutions owned and operated by a government for the benefit of needy persons (contingent upon their financial or medical need), and veterans.
G79	Other Capital Outlay - Welfare, Other	All expenditures for welfare activities not classified elsewhere (for example, expenditures supported by Federal Social Services Block Grant (Title XX) funds)
J67	Assistance and Subsidies – Public Welfare, Federal Categorical Assistance Programs	Includes SSI, TANF, and Medicaid
J68	Assistance and Subsidies – Public Welfare, Cash Assistance Programs, Other	Cash payments made directly to individuals contingent upon their need, other than those under Federal categorical assistance programs.

© 2023 Barnes JM et al. *JAMA Network Open.*

M67	Intergovernmental to Local NEC - Welfare, Categorical Assistance Programs	Includes SSI, TANF, and Medicaid
M68	Intergovernmental to Local NEC - Welfare, Cash Assistance Programs	Cash payments made directly to individuals contingent upon their need, other than those<br>under Federal categorical assistance programs.
M79	Intergovernmental to Local NEC - Welfare	All expenditures for welfare activities not classified elsewhere (for example, expenditures supported by Federal Social Services Block Grant (Title XX) funds)

© 2023 Barnes JM et al. *JAMA Network Open.*

eTable 2: Characteristics of missing/excluded data, 2007-2013

	State Public Assistance expenditures:	Total	≤ $1640 / capita	$1641-1760 / capita	> $1760 / capita
Subgroups		No. (%)			
Age	18-39 years	10578 (4.9)	3937 (5.8)	3146 (4.7)	3495 (4.4)
	40-54 years	35604 (16.6)	12028 (17.6)	10773 (16.1)	12803 (16.2)
	55-64 years	50870 (23.7)	16306 (23.9)	15768 (23.6)	18796 (23.7)
	>65 years	117273 (54.7)	35988 (52.7)	37234 (55.6)	44051 (55.7)
Race and ethnicity	Non-Hispanic White	145179 (67.7)	50267 (73.6)	42474 (63.5)	52438 (66.3)
	Non-Hispanic Black	20914 (9.8)	9848 (14.4)	5307 (7.9)	5759 (7.3)
	Non-Hispanic Other	27323 (12.7)	5262 (7.7)	10776 (16.1)	11285 (14.3)
	Hispanic	20909 (9.8)	2882 (4.2)	8364 (12.5)	9663 (12.2)
Sex	Male	127138 (59.3)	41357 (60.6)	40440 (60.4)	45341 (57.3)
	Female	87187 (40.7)	26902 (39.4)	26481 (39.6)	33804 (42.7)
Residence	Non-metropolitan	25444 (11.9)	11114 (16.3)	5742 (8.6)	8588 (10.9)
	Metropolitan	186410 (87)	57145 (83.7)	61179 (91.4)	68086 (86)
Median household county income (inflation adjusted)	Quartile 1 (low)	47855 (22.3)	21877 (32)	11441 (17.1)	14537 (18.4)
	Quartile 2	56291 (26.3)	11560 (16.9)	21587 (32.3)	23144 (29.2)
	Quartile 3	45863 (21.4)	18132 (26.6)	13959 (20.9)	13772 (17.4)
	Quartile 4 (high)	63599 (29.7)	16685 (24.4)	19911 (29.8)	27003 (34.1)
Marital status	Not married	16764 (7.8)	4315 (6.3)	4980 (7.4)	7469 (9.4)
	Married	8577 (4)	2145 (3.1)	2345 (3.5)	4087 (5.2)
Stage at diagnosis	Localized	112072 (52.3)	39331 (57.6)	33859 (50.6)	38882 (49.1)
	Regional	21806 (10.2)	5858 (8.6)	6869 (10.3)	9079 (11.5)
	Distant	35385 (16.5)	10326 (15.1)	11069 (16.5)	13990 (17.7)
	Unknown/unstaged	45062 (21)	12744 (18.7)	15124 (22.6)	17194 (21.7)
Cancer site	Breast	19179 (8.9)	5042 (7.4)	5986 (8.9)	8151 (10.3)
	Cervical	1456 (0.7)	470 (0.7)	461 (0.7)	525 (0.7)
	Colorectal	14143 (6.6)	4249 (6.2)	4384 (6.6)	5510 (7)
	Head and Neck	6068 (2.8)	1697 (2.5)	1928 (2.9)	2443 (3.1)
	Hodgkin Lymphoma	776 (0.4)	202 (0.3)	274 (0.4)	300 (0.4)
	Kidney and Renal Pelvis	4389 (2)	1183 (1.7)	1274 (1.9)	1932 (2.4)
	Leukemia	7537 (3.5)	2205 (3.2)	2404 (3.6)	2928 (3.7)
	Liver	3322 (1.5)	898 (1.3)	1065 (1.6)	1359 (1.7)
	Lung and Bronchus	17030 (7.9)	5044 (7.4)	4911 (7.3)	7075 (8.9)
	Myeloma	2805 (1.3)	798 (1.2)	895 (1.3)	1112 (1.4)
	Non-Hodgkin Lymphoma	8337 (3.9)	2432 (3.6)	2782 (4.2)	3123 (3.9)
	Other	20950 (9.8)	6401 (9.4)	6331 (9.5)	8218 (10.4)
	Ovary	1890 (0.9)	508 (0.7)	584 (0.9)	798 (1)

© 2023 Barnes JM et al. *JAMA Network Open.*

	Pancreas	4115 (1.9)	1159 (1.7)	1234 (1.8)	1722 (2.2)
	Prostate	53603 (25)	17617 (25.8)	17900 (26.7)	18086 (22.9)
	Skin	29676 (13.8)	13124 (19.2)	8574 (12.8)	7978 (10.1)
	Stomach	2531 (1.2)	702 (1)	795 (1.2)	1034 (1.3)
	Testis	987 (0.5)	260 (0.4)	338 (0.5)	389 (0.5)
	Thyroid	4045 (1.9)	966 (1.4)	1209 (1.8)	1870 (2.4)
	Urinary Bladder	7392 (3.4)	2311 (3.4)	2238 (3.3)	2843 (3.6)
	Uterus	4094 (1.9)	991 (1.5)	1354 (2)	1749 (2.2)
	Mean (standard deviation)				
State Public Welfare	Spending ($) per capita	1686.7 (303.4)	1355.5 (168.2)	1690.7 (40.3)	1968.9 (221.9)

© 2023 Barnes JM et al. *JAMA Network Open.*

eTable 3: Characteristics of the study population, 2007-2019

	State Public Assistance expenditures:	Total	≤ $1640 / capita	$1641-1760 / capita	> $1760 / capita
Subgroup		No. (%)			
Age	18-39 years	223228 (5.8)	60326 (5.9)	46925 (5.7)	115977 (5.7)
	40-54 years	703648 (18.1)	193489 (19)	154817 (18.7)	355342 (17.5)
	55-64 years	1005593 (25.9)	270122 (26.5)	210537 (25.4)	524934 (25.8)
	>65 years	1948598 (50.2)	493828 (48.5)	417261 (50.3)	1037509 (51)
Race and ethnicity	Non-Hispanic White	2684361 (69.2)	740599 (72.8)	574437 (69.2)	1369325 (67.3)
	Non-Hispanic Black	399262 (10.3)	170323 (16.7)	63845 (7.7)	165094 (8.1)
	Non-Hispanic Other	335139 (8.6)	51128 (5)	84555 (10.2)	199456 (9.8)
	Hispanic	462305 (11.9)	55715 (5.5)	106703 (12.9)	299887 (14.7)
Sex	Male	1928698 (49.7)	517256 (50.8)	413975 (49.9)	997467 (49)
	Female	1952369 (50.3)	500509 (49.2)	415565 (50.1)	1036295 (51)
Residence	Non-metropolitan	475756 (12.3)	163402 (16.1)	77976 (9.4)	234378 (11.5)
	Metropolitan	3405311 (87.7)	854363 (83.9)	751564 (90.6)	1799384 (88.5)
Median household county income (inflation adjusted)	Quartile 1 (low)	904088 (23.3)	312838 (30.7)	142564 (17.2)	448686 (22.1)
	Quartile 2	854117 (22)	176235 (17.3)	224492 (27.1)	453390 (22.3)
	Quartile 3	925369 (23.8)	302239 (29.7)	180076 (21.7)	443054 (21.8)
	Quartile 4 (high)	1197493 (30.9)	226453 (22.3)	282408 (34)	688632 (33.9)
Marital status	Not married	1604885 (41.4)	411176 (40.4)	342408 (41.3)	851301 (41.9)
	Married	2276182 (58.6)	606589 (59.6)	487132 (58.7)	1182461 (58.1)
Stage at diagnosis	Localized	1811853 (46.7)	481820 (47.3)	385196 (46.4)	944837 (46.5)
	Regional	855069 (22)	224002 (22)	182140 (22)	448927 (22.1)
	Distant	986267 (25.4)	260942 (25.6)	209501 (25.3)	515824 (25.4)
	Unknown/unstaged	227878 (5.9)	51001 (5)	52703 (6.4)	124174 (6.1)
Cancer site	Breast	622514 (16)	162809 (16)	130871 (15.8)	328834 (16.2)
	Cervical	37179 (1)	9597 (0.9)	8218 (1)	19364 (1)
	Colorectal	329776 (8.5)	85712 (8.4)	72713 (8.8)	171351 (8.4)
	Head and Neck	128807 (3.3)	35193 (3.5)	26678 (3.2)	66936 (3.3)
	Hodgkin Lymphoma	22233 (0.6)	6096 (0.6)	4749 (0.6)	11388 (0.6)

© 2023 Barnes JM et al. *JAMA Network Open.*

	Kidney and Renal Pelvis	137307 (3.5)	34906 (3.4)	28013 (3.4)	74388 (3.7)
	Leukemia	98318 (2.5)	25417 (2.5)	21092 (2.5)	51809 (2.5)
	Liver	73860 (1.9)	16767 (1.6)	16279 (2)	40814 (2)
	Lung and Bronchus	446146 (11.5)	123037 (12.1)	95847 (11.6)	227262 (11.2)
	Myeloma	58682 (1.5)	15925 (1.6)	11784 (1.4)	30973 (1.5)
	Non-Hodgkin Lymphoma	160609 (4.1)	40894 (4)	34474 (4.2)	85241 (4.2)
	Other	413632 (10.7)	105053 (10.3)	88531 (10.7)	220048 (10.8)
	Ovary	57023 (1.5)	14725 (1.4)	12755 (1.5)	29543 (1.5)
	Pancreas	111344 (2.9)	27842 (2.7)	23071 (2.8)	60431 (3)
	Prostate	525384 (13.5)	153076 (15)	114141 (13.8)	258167 (12.7)
	Skin	155639 (4)	38914 (3.8)	33669 (4.1)	83056 (4.1)
	Stomach	62818 (1.6)	14536 (1.4)	13929 (1.7)	34353 (1.7)
	Testis	26869 (0.7)	6645 (0.7)	5823 (0.7)	14401 (0.7)
	Thyroid	125298 (3.2)	30707 (3)	25716 (3.1)	68875 (3.4)
	Urinary Bladder	149162 (3.8)	37171 (3.7)	32663 (3.9)	79328 (3.9)
	Uterus	138467 (3.6)	32743 (3.2)	28524 (3.4)	77200 (3.8)
	Mean (standard deviation)				
State Public Welfare	Spending ($) per capita	1967.0 (621.3)	1317.1 (161.8)	1692.4 (41.4)	2404.3 (538.1)

© 2023 Barnes JM et al. *JAMA Network Open.*

eTable 4: Characteristics of missing/excluded data, 2007-2019

	State Public Assistance expenditures:	Total	≤ $1640 / capita	$1641-1760 / capita	> $1760 / capita
		No. (%)			
Age	18-39 years	21806 (4.9)	6535 (5.6)	4193 (4.9)	11078 (4.5)
	40-54 years	67822 (15.2)	19376 (16.7)	13567 (15.9)	34879 (14.2)
	55-64 years	107489 (24.1)	28273 (24.4)	20235 (23.8)	58981 (24.1)
	>65 years	248977 (55.8)	61732 (53.3)	47099 (55.3)	140146 (57.2)
Race and ethnicity	Non-Hispanic White	288451 (64.7)	85494 (73.8)	56235 (66.1)	146722 (59.9)
	Non-Hispanic Black	43042 (9.6)	18148 (15.7)	6541 (7.7)	18353 (7.5)
	Non-Hispanic Other	68586 (15.4)	7593 (6.6)	13051 (15.3)	47942 (19.6)
	Hispanic	46015 (10.3)	4681 (4)	9267 (10.9)	32067 (13.1)
Sex	Male	264340 (59.3)	70040 (60.4)	50557 (59.4)	143743 (58.7)
	Female	181754 (40.7)	45876 (39.6)	34537 (40.6)	101341 (41.3)
Residence	Non-metropolitan	52274 (11.7)	18807 (16.2)	7094 (8.3)	26373 (10.8)
	Metropolitan	388922 (87.2)	97109 (83.8)	78000 (91.7)	213813 (87.2)
Median household county income (inflation adjusted)	Quartile 1 (low)	98916 (22.2)	37304 (32.2)	12362 (14.5)	49250 (20.1)
	Quartile 2	100870 (22.6)	20603 (17.8)	23870 (28.1)	56397 (23)
	Quartile 3	102297 (22.9)	31743 (27.4)	18934 (22.3)	51620 (21.1)
	Quartile 4 (high)	143103 (32.1)	26252 (22.6)	29896 (35.1)	86955 (35.5)
Marital status	Not married	38789 (8.7)	7706 (6.6)	5838 (6.9)	25245 (10.3)
	Married	18886 (4.2)	3740 (3.2)	2664 (3.1)	12482 (5.1)
Stage at diagnosis	Localized	220452 (49.4)	65832 (56.8)	43991 (51.7)	110629 (45.1)
	Regional	43951 (9.9)	9972 (8.6)	8983 (10.6)	24996 (10.2)
	Distant	67785 (15.2)	17350 (15)	13812 (16.2)	36623 (14.9)
	Unknown/unstaged	113906 (25.5)	22762 (19.6)	18308 (21.5)	72836 (29.7)
Cancer site	Breast	39734 (8.9)	8419 (7.3)	8017 (9.4)	23298 (9.5)
	Cervical	2950 (0.7)	774 (0.7)	583 (0.7)	1593 (0.6)
	Colorectal	28662 (6.4)	7083 (6.1)	5626 (6.6)	15953 (6.5)
	Head and Neck	12540 (2.8)	3005 (2.6)	2518 (3)	7017 (2.9)
	Hodgkin Lymphoma	1463 (0.3)	341 (0.3)	331 (0.4)	791 (0.3)
	Kidney and Renal Pelvis	9288 (2.1)	1999 (1.7)	1695 (2)	5594 (2.3)
	Leukemia	14592 (3.3)	3616 (3.1)	2925 (3.4)	8051 (3.3)
	Liver	7740 (1.7)	1407 (1.2)	1336 (1.6)	4997 (2)
	Lung and Bronchus	34953 (7.8)	8779 (7.6)	5991 (7)	20183 (8.2)
	Myeloma	5710 (1.3)	1380 (1.2)	1095 (1.3)	3235 (1.3)
	Non-Hodgkin Lymphoma	16262 (3.6)	3809 (3.3)	3455 (4.1)	8998 (3.7)
	Other	43732 (9.8)	11128 (9.6)	7988 (9.4)	24616 (10)

© 2023 Barnes JM et al. *JAMA Network Open.*

	Ovary	3735 (0.8)	836 (0.7)	721 (0.8)	2178 (0.9)
	Pancreas	9369 (2.1)	1974 (1.7)	1544 (1.8)	5851 (2.4)
	Prostate	108128 (24.2)	28950 (25)	21656 (25.4)	57522 (23.5)
	Skin	65922 (14.8)	23172 (20)	11451 (13.5)	31299 (12.8)
	Stomach	5270 (1.2)	1175 (1)	999 (1.2)	3096 (1.3)
	Testis	2241 (0.5)	457 (0.4)	479 (0.6)	1305 (0.5)
	Thyroid	8570 (1.9)	1671 (1.4)	1691 (2)	5208 (2.1)
	Urinary Bladder	15982 (3.6)	4058 (3.5)	3160 (3.7)	8764 (3.6)
	Uterus	9251 (2.1)	1883 (1.6)	1833 (2.2)	5535 (2.3)
	Mean (standard deviation)				
State Public Welfare	Spending ($) per capita	1992.3 (634.9)	1310.8 (149.7)	1698.8 (41.5)	2416.5 (541.3)

© 2023 Barnes JM et al. *JAMA Network Open.*

eTable 5: State fixed effects for primary analysis (overall cohort, 2007-2013)

	6-year overall survival difference, % (95% CI)	P-value
California	Reference	Reference
Connecticut	0.67 (-2.84, 4.18)	0.708
Georgia	0.21 (-1.01, 1.44)	0.733
Hawaii	-3.53 (-6.81, -0.24)	0.035
Iowa	-1.8 (-3.98, 0.37)	0.105
Kentucky	-4.46 (-5.82, -3.09)	0
Louisiana	-3.39 (-4.81, -1.97)	0
New Jersey	0.26 (-2.97, 3.49)	0.875
New Mexico	-4.82 (-7.15, -2.5)	0
Utah	4.12 (2.87, 5.37)	0
Washington	1.33 (-0.02, 2.67)	0.053

© 2023 Barnes JM et al. *JAMA Network Open.*

eTable 6: Sensitivity analysis evaluating associations of public assistance spending and 6-year cancer-specific survival (CSS), 2007-2013

	2007-2013, 6-yr CSS (%)	
	Estimate (95% CI)	P-value
Overall	0.06 (0.01, 0.1)	0.017
Non-Hispanic White	0.09 (0.04, 0.14)	0
Non-Hispanic Black	0.23 (0.04, 0.41)	0.015
Non-Hispanic Other	-0.09 (-0.23, 0.05)	0.213
Hispanic	0.02 (-0.09, 0.12)	0.713

© 2023 Barnes JM et al. *JAMA Network Open.*

eTable 7: Association of components of public assistance expenditures and 6-year overall survival

	Subgroup			
Component	Age	Race and Ethnicity	Estimate (95% CI)[a,b]	P-value
Total Public Assistance Expenditures	18-64 years	Overall	0.07 (0.03, 0.1)	<.001
		Non-Hispanic White	0.13 (0.1, 0.16)	<.001
		Non-Hispanic Black	0.04 (0.01, 0.07)	0.017
		Non-Hispanic Other	-0.07 (-0.14, 0.01)	0.1
		Hispanic	0.07 (0.02, 0.13)	0.012
	>65 years	Overall	0.03 (0.01, 0.05)	0.002
		Non-Hispanic White	0.08 (0.04, 0.11)	<.001
		Non-Hispanic Black	-0.09 (-0.15, -0.03)	0.004
		Non-Hispanic Other	0.07 (-0.01, 0.15)	0.077
		Hispanic	0.02 (-0.05, 0.09)	0.509
Supplemental Security Income Expenditures	Overall Sample	Overall	4.07 (-18.92, 27.07)	0.728
		Non-Hispanic White	-4.17 (-23.34, 14.99)	0.669
		Non-Hispanic Black	5.52 (-32.75, 43.79)	0.777
		Non-Hispanic Other	4.42 (-22.21, 31.04)	0.745
		Hispanic	-13.03 (-49.14, 23.09)	0.48
	18-64 years	Overall	-0.73 (-36.08, 34.61)	0.968
		Non-Hispanic White	-5.92 (-38.6, 26.76)	0.722
		Non-Hispanic Black	-0.37 (-70.76, 70.02)	0.992
		Non-Hispanic Other	4.89 (-42.12, 51.91)	0.838
		Hispanic	-21.57 (-43.57, 0.44)	0.055
	>65 years	Overall	-4.41 (-26.73, 17.92)	0.699
		Non-Hispanic White	-13.45 (-33.86, 6.97)	0.197
		Non-Hispanic Black	2.84 (-61.29, 66.98)	0.931
		Non-Hispanic Other	4.48 (-18.18, 27.14)	0.698
		Hispanic	-14.55 (-43.19, 14.08)	0.319
Temporary Assistance for Needy Families Expenditures	Overall Sample	Overall	-0.21 (-0.73, 0.3)	0.411
		Non-Hispanic White	-0.12 (-0.49, 0.25)	0.523
		Non-Hispanic Black	-0.22 (-2.08, 1.65)	0.821
		Non-Hispanic Other	-0.79 (-1.54, -0.05)	0.036
		Hispanic	-1.01 (-2.96, 0.93)	0.308
	18-64 years	Overall	-0.86 (-1.58, -0.14)	0.019
		Non-Hispanic White	-1.14 (-2.1, -0.17)	0.021
		Non-Hispanic Black	-0.22 (-1.98, 1.54)	0.808
		Non-Hispanic Other	-1.56 (-2.32, -0.8)	<.001
		Hispanic	0.32 (-0.85, 1.49)	0.595

© 2023 Barnes JM et al. *JAMA Network Open.*

	>65 years[c]	Overall	NA	NA
		Non-Hispanic White	NA	NA
		Non-Hispanic Black	NA	NA
		Non-Hispanic Other	NA	NA
		Hispanic	NA	NA
Medicaid Expenditures	Overall Sample	Overall	0.04 (0.01, 0.06)	0.01
		Non-Hispanic White	0.03 (-0.01, 0.06)	0.105
		Non-Hispanic Black	0.16 (0.06, 0.27)	0.002
		Non-Hispanic Other	0.02 (-0.08, 0.11)	0.717
		Hispanic	0 (-0.13, 0.14)	0.983
	18-64 years	Overall	0.04 (0, 0.09)	0.066
		Non-Hispanic White	0.09 (0.04, 0.13)	<.001
		Non-Hispanic Black	0.03 (-0.08, 0.14)	0.575
		Non-Hispanic Other	-0.03 (-0.09, 0.04)	0.415
		Hispanic	0 (-0.02, 0.03)	0.875
	>65 years	Overall	0.03 (-0.02, 0.08)	0.263
		Non-Hispanic White	0.04 (-0.03, 0.11)	0.223
		Non-Hispanic Black	-0.05 (-0.23, 0.12)	0.546
		Non-Hispanic Other	0.07 (0, 0.15)	0.05
		Hispanic	0.01 (-0.06, 0.07)	0.823
Other Expenditures	Overall Sample	Overall	0.14 (0.05, 0.23)	0.003
		Non-Hispanic White	0.19 (0.09, 0.29)	<.001
		Non-Hispanic Black	0.17 (-0.16, 0.49)	0.314
		Non-Hispanic Other	-0.11 (-0.36, 0.13)	0.358
		Hispanic	-0.02 (-0.2, 0.16)	0.793
	18-64 years	Overall	0.04 (-0.1, 0.19)	0.557
		Non-Hispanic White	0.08 (-0.1, 0.25)	0.38
		Non-Hispanic Black	-0.01 (-0.12, 0.11)	0.925
		Non-Hispanic Other	-0.08 (-0.22, 0.07)	0.292
		Hispanic	0.13 (-0.01, 0.27)	0.075
	>65 years	Overall	-0.01 (-0.15, 0.13)	0.847
		Non-Hispanic White	0.04 (-0.12, 0.19)	0.664
		Non-Hispanic Black	-0.13 (-0.26, 0.01)	0.069
		Non-Hispanic Other	-0.03 (-0.35, 0.3)	0.862
		Hispanic	-0.05 (-0.16, 0.06)	0.33

[a]Estimates are scaled to change in survival per $100/capita.
[b]Models were adjusted for age (except age subgroup analyses), race and ethnicity (except race and ethnicity subgroup analyses), sex, metropolitan residence, marital status, county-level income, state fixed effects, state-level poverty and % elderly, cancer type, and cancer stage.
[c]TANF spending was not included as a component of public assistance spending for the >65 year subgroup analyses given that TANF spending is focused primarily on younger individuals and families.

© 2023 Barnes JM et al. *JAMA Network Open.*

eTable 8: Subgroup analyses by cancer site and county income level (overall cohort, 2007-2013)

Subgroup		6-yr overall survival difference, % (95% CI)	P-value
Cancer site	Breast	0.06 (-0.02, 0.14)	0.151
	Cervical	0.03 (-0.18, 0.23)	0.789
	Colorectal	-0.13 (-0.26, -0.01)	0.04
	Head and Neck	0.33 (0.18, 0.48)	0
	Hodgkin Lymphoma	0.29 (-0.33, 0.9)	0.357
	Kidney and Renal Pelvis	0.28 (0.13, 0.43)	0
	Leukemia	0.45 (0.07, 0.83)	0.021
	Liver	0.39 (0.23, 0.55)	0
	Lung and Bronchus	0.05 (-0.01, 0.11)	0.082
	Myeloma	0.67 (0.32, 1.03)	0
	Non-Hodgkin Lymphoma	0.32 (0.11, 0.54)	0.003
	Other	-0.2 (-0.4, 0)	0.05
	Ovary	0.23 (-0.07, 0.53)	0.133
	Pancreas	0.05 (-0.11, 0.2)	0.538
	Prostate	0.21 (0.13, 0.3)	0
	Skin[a]	0.29 (0.14, 0.44)	0
	Stomach	0.12 (-0.21, 0.45)	0.475
	Testis	0.3 (0.08, 0.52)	0.008
	Thyroid	-0.02 (-0.13, 0.08)	0.647
	Urinary Bladder	-0.05 (-0.35, 0.25)	0.739
	Uterus	0.23 (0.12, 0.35)	0
County income[b]	Lowest quartile	0.1 (0.02, 0.18)	0.017
	Second quartile	0.04 (-0.04, 0.12)	0.29
	Third quartile	0.19 (0.13, 0.24)	0
	Highest quartile	0.07 (0.03, 0.12)	0.003

[a] Includes melanoma and non-melanoma skin cancers, but excludes cutaneous basal and squamous cell carcinoma, which are not included in SEER.
[b] We also tested for the presence of an interaction between public assistance spending and county income, but these were not statistically significant (P>.16).

© 2023 Barnes JM et al. *JAMA Network Open.*

eTable 9: Sensitivity analyses adjusting for cost of living by state and for state Medicaid eligibility levels (2007-2013)

			Overall		<65 years		≥65 years	
Sensitivity Analysis	Component of spending	Subgroup	Estimate (95% CI)	P-value	Estimate (95% CI)	P-value	Estimate (95% CI)	P-value
Adjust for cost of living	Total Social Spending	Overall	0.13 (0.07, 0.18)	0	0.09 (-0.01, 0.2)	0.075	0.15 (0.1, 0.2)	0
		Non-Hispanic White	0.16 (0.1, 0.22)	0	0.15 (0.06, 0.24)	0.001	0.15 (0.06, 0.23)	0.001
		Non-Hispanic Black	0.37 (0.12, 0.61)	0.004	0.41 (0.04, 0.77)	0.029	0.29 (0.02, 0.56)	0.036
		Non-Hispanic Other	-0.05 (-0.24, 0.13)	0.57	-0.22 (-0.53, 0.09)	0.173	0.14 (-0.11, 0.39)	0.276
		Hispanic	0.09 (-0.03, 0.21)	0.128	0 (-0.19, 0.19)	0.996	0.24 (0.06, 0.41)	0.007
	SSIP	Overall	13.37 (-11.25, 37.98)	0.287	20.83 (-2.03, 43.7)	0.074	4.34 (-21.89, 30.57)	0.746
		Non-Hispanic White	4.12 (-16.72, 24.96)	0.699	12.09 (-2.58, 26.77)	0.106	-3.44 (-28.16, 21.29)	0.785
		Non-Hispanic Black	17.62 (-28.38, 63.62)	0.453	35.87 (-33.3, 105.04)	0.309	-11.69 (-46.73, 23.34)	0.513
		Non-Hispanic Other	-4.32 (-38.02, 29.38)	0.802	-18.58 (-48.38, 11.21)	0.222	16.18 (-65.24, 97.59)	0.697
		Hispanic	-13.11 (-56.74, 30.52)	0.556	-6.37 (-50.31, 37.57)	0.776	-18.44 (-69.08, 32.2)	0.475
	TANFp	Overall	-0.2 (-0.64, 0.23)	0.361	-0.03 (-0.69, 0.63)	0.925		
		Non-Hispanic White	0.03 (-0.29, 0.36)	0.845	0.22 (-0.06, 0.5)	0.124		
		Non-Hispanic Black	-0.17 (-2.25, 1.92)	0.876	0.88 (-3.65, 5.4)	0.705		
		Non-Hispanic Other	-1.67 (-3.07, -0.28)	0.019	-2.56 (-4.37, -0.75)	0.006		
		Hispanic	-1.86 (-4.05, 0.34)	0.097	-0.89 (-4.38, 2.6)	0.618		
	Medicaid	Overall	-0.01 (-0.05, 0.02)	0.404	-0.02 (-0.07, 0.04)	0.573	-0.01 (-0.06, 0.05)	0.831
		Non-Hispanic White	-0.04 (-0.08, -0.01)	0.019	-0.06 (-0.12, 0.01)	0.072	-0.03 (-0.1, 0.05)	0.511
		Non-Hispanic Black	0.1 (-0.03, 0.23)	0.124	0.05 (-0.12, 0.23)	0.547	0.19 (0.05, 0.34)	0.009
		Non-Hispanic Other	0.11 (-0.04, 0.25)	0.141	0.15 (-0.02, 0.32)	0.077	0.05 (-0.13, 0.24)	0.575

© 2023 Barnes JM et al. *JAMA Network Open.*

		Hispanic	0.05 (-0.13, 0.23)	0.568	0.11 (-0.25, 0.48)	0.549	-0.06 (-0.3, 0.18)	0.628
	Other spending	Overall	0.17 (0.11, 0.24)	0	0.16 (0.08, 0.24)	0	0.17 (0.08, 0.26)	0
		Non-Hispanic White	0.23 (0.15, 0.3)	0	0.26 (0.17, 0.35)	0	0.18 (0.04, 0.33)	0.014
		Non-Hispanic Black	0.26 (-0.04, 0.55)	0.088	0.42 (-0.04, 0.88)	0.073	-0.03 (-0.36, 0.29)	0.84
		Non-Hispanic Other	-0.22 (-0.51, 0.08)	0.152	-0.48 (-0.95, -0.01)	0.045	0.11 (-0.37, 0.59)	0.646
		Hispanic	-0.02 (-0.25, 0.22)	0.892	-0.16 (-0.51, 0.2)	0.387	0.24 (-0.07, 0.55)	0.127
Adjust for Medicaid eligibility levels[a]	Total Social Spending	Overall	0.09 (0.04, 0.13)	0	0.06 (-0.02, 0.13)	0.136	0.12 (0.09, 0.15)	0
		Non-Hispanic White	0.12 (0.08, 0.17)	0	0.12 (0.05, 0.18)	0.001	0.12 (0.07, 0.17)	0
		Non-Hispanic Black	0.28 (0.07, 0.5)	0.009	0.29 (-0.03, 0.61)	0.08	0.25 (0.08, 0.43)	0.003
		Non-Hispanic Other	-0.01 (-0.16, 0.15)	0.94	-0.11 (-0.29, 0.06)	0.211	0.12 (-0.1, 0.34)	0.277
		Hispanic	0.05 (-0.03, 0.13)	0.221	-0.02 (-0.13, 0.09)	0.687	0.18 (0.06, 0.3)	0.004
	SSIP	Overall	8.88 (-18.75, 36.51)	0.529	15.46 (-10.55, 41.47)	0.244	1.28 (-27.66, 30.21)	0.931
		Non-Hispanic White	1.88 (-19.7, 23.47)	0.864	10.58 (-4.54, 25.69)	0.17	-6.04 (-32.16, 20.08)	0.65
		Non-Hispanic Black	10.37 (-39.87, 60.61)	0.686	24.46 (-54.3, 103.23)	0.543	-13.61 (-54.85, 27.63)	0.518
		Non-Hispanic Other	0 (-37.95, 37.96)	1	-1.97 (-24.19, 20.26)	0.862	1.14 (-66.81, 69.09)	0.974
		Hispanic	-15.14 (-59.61, 29.34)	0.505	-19.14 (-57.98, 19.7)	0.334	-4.51 (-60.66, 51.63)	0.875
	TANFp	Overall	-0.05 (-0.6, 0.49)	0.845	0.04 (-0.7, 0.78)	0.92		
		Non-Hispanic White	0.11 (-0.24, 0.47)	0.538	0.28 (-0.01, 0.56)	0.056		
		Non-Hispanic Black	0.15 (-1.84, 2.13)	0.886	0.76 (-2.94, 4.46)	0.687		
		Non-Hispanic Other	-1.08 (-1.83, -0.34)	0.004	-1.73 (-2.64, -0.83)	0		
		Hispanic	-1.22 (-3.3, 0.86)	0.251	-0.4 (-3.3, 2.49)	0.785		
	Medicaid[a]	Overall	-0.01 (-0.05, 0.02)	0.364	-0.02 (-0.07, 0.04)	0.527	-0.01 (-0.05, 0.04)	0.74

© 2023 Barnes JM et al. *JAMA Network Open.*

		Non-Hispanic White	-0.04 (-0.07, -0.01)	0.02	-0.06 (-0.12, 0)	0.056	-0.02 (-0.09, 0.05)	0.531
		Non-Hispanic Black	0.11 (-0.03, 0.24)	0.133	0.07 (-0.11, 0.26)	0.422	0.17 (-0.01, 0.35)	0.064
		Non-Hispanic Other	0.08 (0, 0.17)	0.062	0.11 (-0.01, 0.24)	0.073	0.04 (-0.07, 0.16)	0.469
		Hispanic	0.03 (-0.14, 0.19)	0.767	0.05 (-0.28, 0.39)	0.765	-0.04 (-0.24, 0.17)	0.728
	Other spending	Overall	0.14 (0.04, 0.23)	0.004	0.13 (0.02, 0.24)	0.017	0.13 (0.03, 0.23)	0.009
		Non-Hispanic White	0.19 (0.09, 0.29)	0	0.23 (0.14, 0.32)	0	0.14 (-0.01, 0.28)	0.062
		Non-Hispanic Black	0.17 (-0.18, 0.52)	0.334	0.28 (-0.27, 0.83)	0.318	-0.04 (-0.38, 0.3)	0.823
		Non-Hispanic Other	-0.14 (-0.37, 0.1)	0.251	-0.3 (-0.61, 0.01)	0.061	0.06 (-0.25, 0.37)	0.711
		Hispanic	-0.04 (-0.21, 0.12)	0.604	-0.15 (-0.47, 0.16)	0.336	0.17 (-0.13, 0.48)	0.265

[a]Given attenuated Medicaid expenditure effects after accounting for Medicaid eligibility criteria, differential Medicaid eligibility likely drives the associations of Medicaid spending and survival, which is consistent with reports of increased survival associated with Medicaid expansion (i.e. expanded Medicaid eligibility criteria).

© 2023 Barnes JM et al. *JAMA Network Open.*

eFigure 1: Flowchart for derivation of study sample

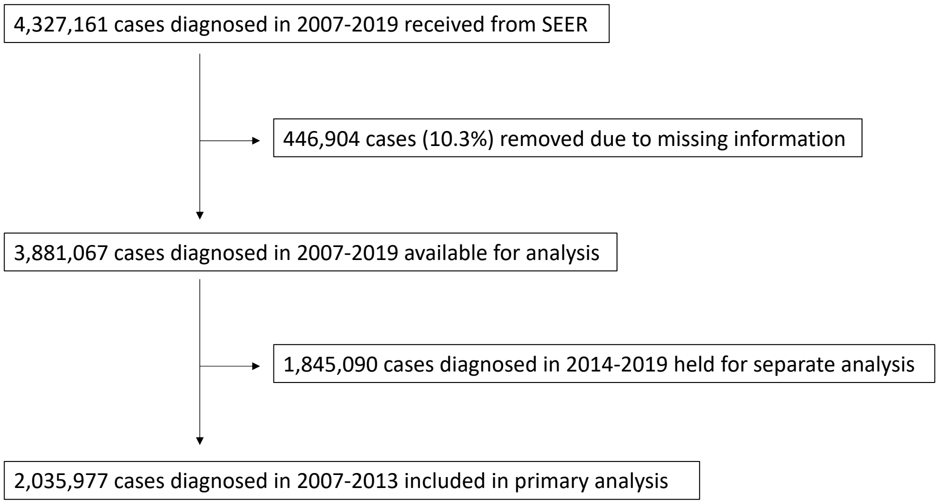

© 2023 Barnes JM et al. *JAMA Network Open.*

eFigure 2: Distribution of public welfare spending across individuals from states included in the Surveillance, Epidemiology, and End Results Program, 2007-2013

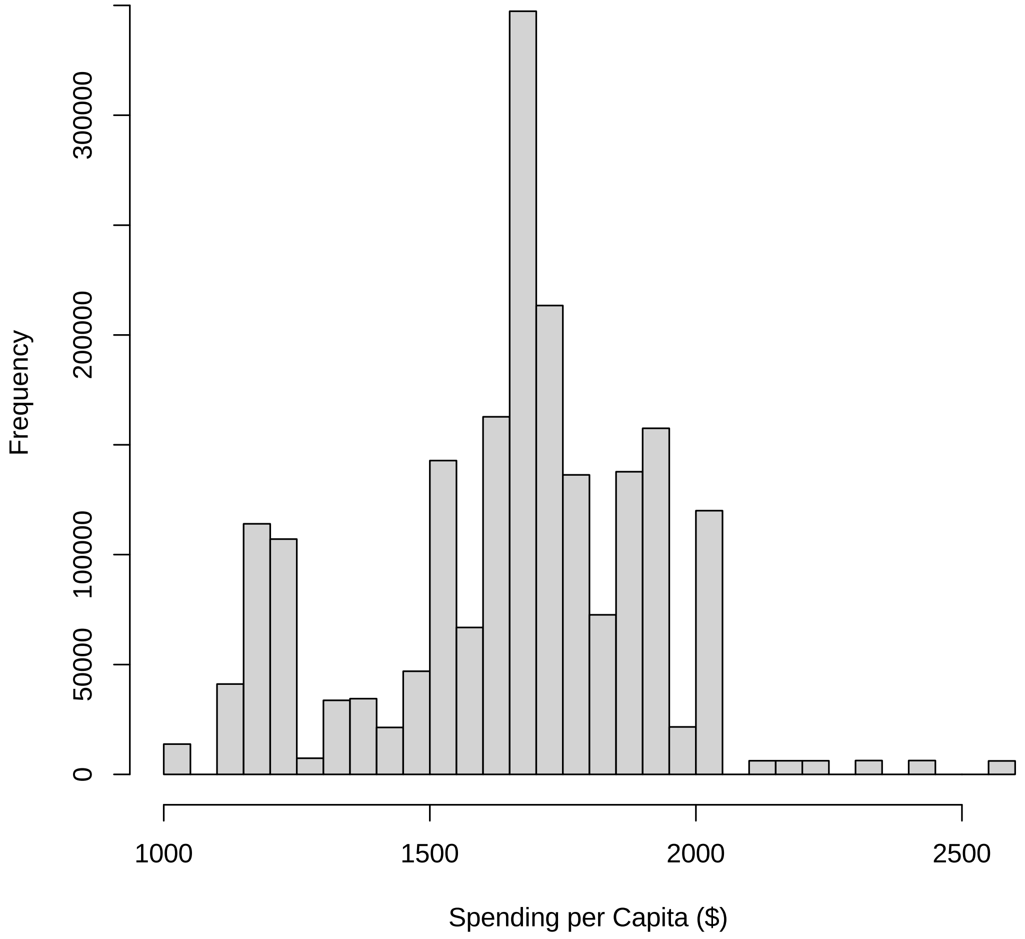

© 2023 Barnes JM et al. *JAMA Network Open.*

eFigure 3: Distribution of public welfare spending across individuals from states included in the Surveillance, Epidemiology, and End Results Program, 2007-2019

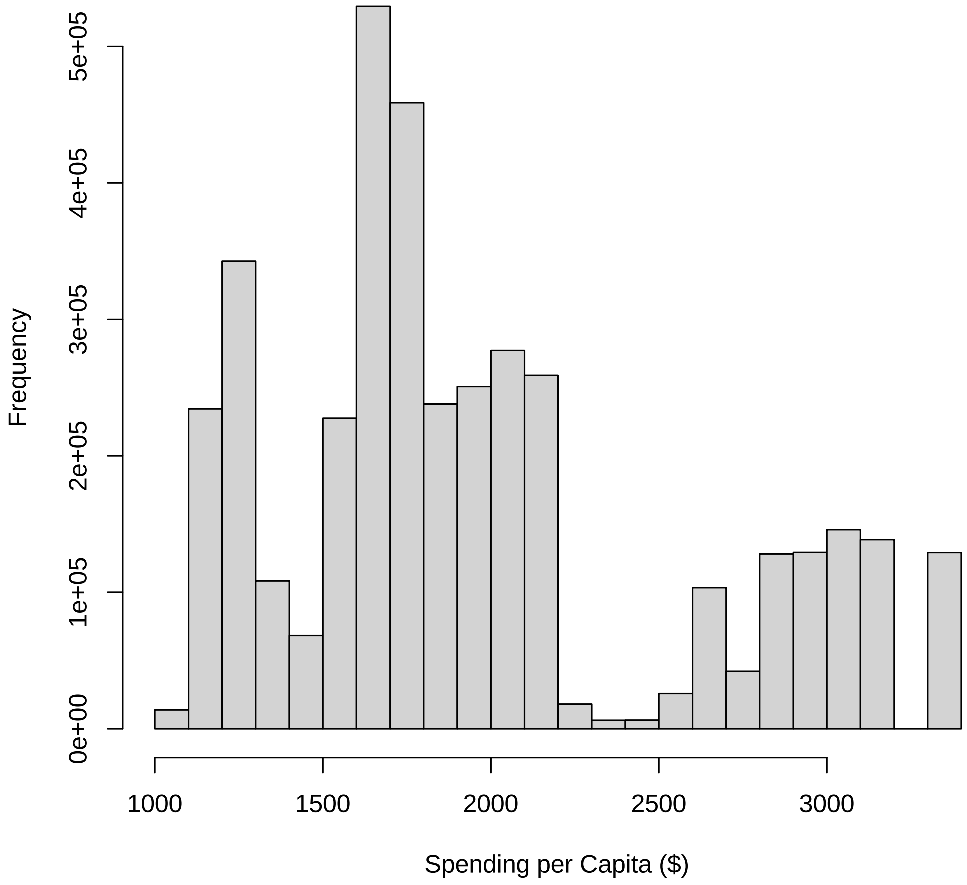

© 2023 Barnes JM et al. *JAMA Network Open.*

eFigure 4: Overall survival by state public assistance expenditures for non-Hispanic Other and Hispanic individuals

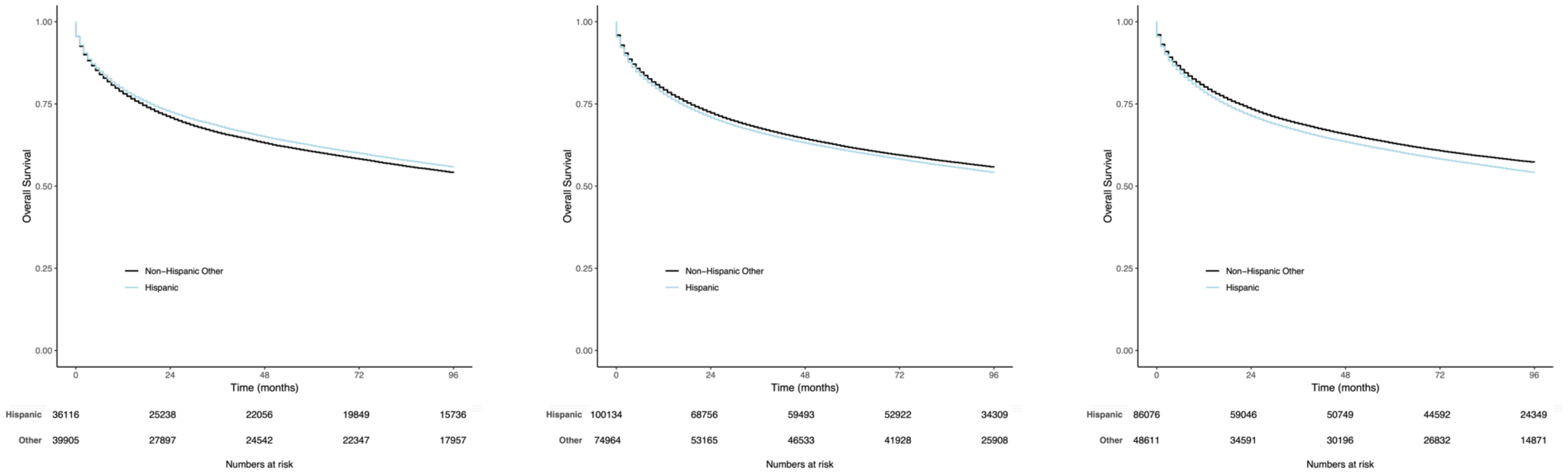

(A) ≤ $1640 per capita

(B) $1640 - 1760 per capita

(C) > $1760 per capita

© 2023 Barnes JM et al. *JAMA Network Open.*
