# Supplementary material for: State Public Assistance Spending and Survival Among Adults With Cancer
Source: JAMA Netw Open. 2023 Sep 5;6(9):e2332353. doi: 10.1001/jamanetworkopen.2023.32353 (PMC10481229; doi:10.1001/jamanetworkopen.2023.32353)
Supplement: Supplement 2. — Data Sharing Statement [file jamanetwopen-e2332353-s002.pdf]

Data Sharing Statement

Barnes. State Public Assistance Spending and Survival Among Adults With Cancer. *JAMA Netw Open*. Published September 05, 2023. doi:10.1001/jamanetworkopen.2023.32353

Data
Data available: No

Additional Information
Explanation for why data not available: Data are already publicly available following request from National Cancer Institute.
